# How have the clinical, laboratory, treatment features and outcomes in children with lupus nephritis progressed over the last 30 years?

**DOI:** 10.1093/rheumatology/keaf151

**Published:** 2025-03-19

**Authors:** Deniz Gezgin Yıldırım, Nihal Karaçayır, Hakan Kısaoğlu, Aydan Yekedüz Bülbül, Pınar Garipçin, Hülya Nalçacıoğlu, Mukaddes Kalyoncu, Hakan Poyrazoğlu, Sevcan A Bakkaloğlu

**Affiliations:** Department of Pediatric Rheumatology, Gazi University Faculty of Medicine, Ankara, Turkey; Department of Pediatric Rheumatology, Gazi University Faculty of Medicine, Ankara, Turkey; Department of Pediatric Rheumatology, Karadeniz Technical University Faculty of Medicine, Trabzon, Turkey; Department of Pediatric Rheumatology, Erciyes University Faculty of Medicine, Kayseri, Turkey; Department of Pediatric Rheumatology, Erciyes University Faculty of Medicine, Kayseri, Turkey; Department of Pediatric Nephrology, Ondokuz Mayıs University Faculty of Medicine, Samsun, Turkey; Department of Pediatric Rheumatology, Karadeniz Technical University Faculty of Medicine, Trabzon, Turkey; Department of Pediatric Nephrology, Karadeniz Technical University Faculty of Medicine, Trabzon, Turkey; Department of Pediatric Rheumatology, Erciyes University Faculty of Medicine, Kayseri, Turkey; Department of Pediatric Nephrology, Erciyes University Faculty of Medicine, Kayseri, Turkey; Department of Pediatric Rheumatology, Gazi University Faculty of Medicine, Ankara, Turkey; Department of Pediatric Nephrology, Gazi University Faculty of Medicine, Ankara, Turkey

**Keywords:** lupus nephritis, outcome, paediatric rheumatology, systemic lupus erythematosus

## Abstract

**Objectives:**

Management of systemic lupus erythematosus (SLE) through new treatment options has improved lupus nephritis (LN) prognosis. The aim of this study was to compare the changes in the demographic, laboratory, and treatment characteristics, prognosis, and outcomes of paediatric-onset LN patients over 30 years.

**Methods:**

We retrospectively reviewed the medical records of 103 paediatric-onset LN patients. Patients were divided into two subgroups according to the years of LN diagnosis. Group 1 consisted of patients diagnosed with LN between the years of 1993 and 2005, and group 2 consisted of patients diagnosed with LN between the years of 2006 and 2023.

**Results:**

The mean age at diagnosis of SLE, age at diagnosis of LN, time to LN development, and mean delay time to diagnosis were significantly higher in group 1 (*P* < 0.001, *P* < 0.001, *P* = 0.049, and *P* = 0.004, respectively). Baseline Systemic Lupus Erythematosus Disease Activity Index (SLEDAI) scores were higher and anti-phospholipid antibody positivity was more frequent in group 1 (*P* = 0.040 and *P* = 0.025, respectively). Azathioprine in the maintenance phase was given more frequently in group 1 (*P* = 0.016), while rituximab was more frequently used in group 2 (*P* = 0.042). In both groups, the majority of the patients had proliferative LN (class III and/or class IV) (53.5% in group 1 vs. 68% in group 2). Complete renal remission was significantly more common in group 2 (*P* = 0.005), while end-stage kidney disease (ESKD) and death were significantly more common in group 1 (*P* = 0.005 and *P* = 0.001, respectively). Proteinuria and SLEDAI scores at the first visit were independent risk factors for progression to ESKD (*P* = 0.037 and *P* = 0.024).

**Conclusion:**

Over the years, there have been significant improvements in the diagnosis and management of children with SLE resulting in an earlier diagnosis, lower disease activity at onset, and improved outcomes.

Rheumatology key messages

- End-stage kidney disease and death related to lupus nephritis (LN) has decreased in the last 15 years.
- Except for anti-phospholipid antibodies positivity, serologic and laboratory features of the paediatric-onset LN patients did not change significantly over a 30-year period.
- Patients with paediatric-onset LN have shown an improved disease outcome due to better management with new treatment modalities, such as rituximab, over recent years.

## Introduction

Paediatric-onset systemic lupus erythematosus (SLE) is a rare but severe autoimmune disease that affects multiple organ systems due to loss of self-tolerance, development of autoantibodies against the cell structures, immune complexes and aberrant activation of the complement system [1]. Paediatric-onset SLE has a more severe disease course and needs a more aggressive treatment strategy compared with adult-onset SLE [2]. Lupus nephritis (LN) is the most common solid organ involvement in SLE that may be the initial manifestation or become manifest at any time of the disease course. Patients with LN may present with various clinical findings ranging from asymptomatic urinary abnormalities to gross haematuria, massive proteinuria and kidney failure [3]. LN develops in 25–80% of patients with paediatric-onset SLE and is responsible for end-stage kidney disease (ESKD) and death [4–6]. Factors associated with the poor outcome and severity of LN include histopathologic classification of LN (proliferative nephritis), male gender, African/Caribbean ethnicity, hypertension, poor treatment response, renal impairment at disease onset and recurrence of LN [7, 8]. The mortality rate of SLE patients with and without LN is ∼7 times and 2 times higher compared with the healthy population [9–11]. Corticosteroids in combination with conventional disease-modifying anti-rheumatic drugs (cDMARDs) including hydroxychloroquine (HCQ), mycophenolate mofetil (MMF), azathioprine (AZA) and cyclophosphamide (CYC) are the mainstay of the treatment in LN of childhood. In recent years, new biologic DMARDs, such as rituximab (RTX) and belimumab, have successfully been used in paediatric-onset SLE [1, 12]. The 5-year survival rate of LN was 50% in the 1960s and improved significantly to 80% in the 1990s [13]. A better understanding of SLE pathogenesis, strict control of hypertension and proteinuria, and better management of cardiovascular risk factors with newly developed drugs are the leading causes of improved LN prognosis [14, 15].

There are comparative data for the prognosis of adult-onset LN between 1990s and 2000s [16, 17]. However, data comparing the long-term prognosis and outcomes of paediatric-onset LN patients are scarce [6, 18, 19]. This study aimed to compare the changes in the demographic, laboratory and treatment characteristics, prognosis, and outcomes of paediatric-onset LN patients treated in the 1990s and 2000s, over the last 30 years.

## Methods

This is a multicentre and case-controlled study. We investigated the medical records of 111 paediatric-onset LN patients who were followed up at the paediatric rheumatology and paediatric nephrology centres of Gazi University Hospital, Erciyes University Hospital, Karadeniz Technical University Hospital and Ondokuz Mayıs University Hospital between the years 1993 and 2023. Paediatric-onset LN diagnosed at younger than 16 years of age and followed up for >6 months were included in the study. Six patients were excluded from the study because of missing data and 2 due to a follow-up time of <6 months. Therefore, 103 patients were eligible for the study (Supplementary Fig. S1, available at *Rheumatology* online).

Diagnosis of SLE was made by American College Rheumatology classification criteria [20]. Renal biopsy was performed on the patients with SLE who had persistent haematuria and/or proteinuria, increased serum creatinine level, or glomerular filtration rate (GFR) <90 ml/min/1.73 m^2^. The International Society of Nephrology/Renal Pathology Society 2003 classification criteria were used in the diagnosis and classification of LN [20]. Accordingly, class I: minimal mesangial LN, class II: mesangial proliferative LN, class III: focal proliferative LN (<50% of glomeruli affected), class IV: diffuse proliferative LN (≥50% of glomeruli affected), class V: membranous LN and class VI: advanced sclerosing LN (≥90% of glomeruli sclerosed) [21]. Systemic Lupus Erythematosus Disease Activity Index (SLEDAI) was used for the assessment of disease activity in all patients at the diagnosis of LN and last visit [22].

Demographics, clinical and laboratory features, kidney biopsy results, given treatments, treatment responses and outcomes were recorded from the medical charts of the patients. Clinical signs, such as fever, myositis, arthritis, alopecia, and cutaneous, neurologic, haematologic, pulmonary and cardiovascular involvement related to SLE were noted. Cardiovascular involvement was accepted as pericarditis, myocarditis, endocarditis, coronary artery disease and hypertension; pulmonary involvement as pleural effusion and/or interstitial lung disease; haematologic involvement as leukopenia, anaemia and/or thrombocytopenia; and neurologic involvement as central or peripheral nervous system diseases. In laboratory studies, blood urea nitrogen (BUN), creatinine, complete blood count, erythrocyte sedimentation rate (ESR), C-reactive protein (CRP), proteinuria, haematuria, and serum complement levels were noted at first and last visits for each patient. We also recorded the antinuclear antibody (ANA), anti-double-stranded DNA (anti-dsDNA) antibody, and antiphospholipid antibodies. An ANA titre of 1:80 was considered positive [23] and was measured by indirect immunofluorescence. Anti-dsDNA antibody was evaluated by ELISA. C3 and C4 levels were evaluated by nephelometry. Hypertension is accepted as systolic or diastolic blood pressure in the 95th percentile and above according to age, height and gender [24].

Regarding disease outcome, complete response (CR) was considered as a normal renal function (GFR > 90 ml/min) and proteinuria <0.5 g/day and partial response (PR) was accepted as a > 50% reduction in proteinuria level. Kidney Disease Improving Global Outcomes classification was used for chronic kidney disease (CKD) stages [25]. ESKD was defined as an eGFR <15 ml/min (CKD stage 5) or those undergoing chronic dialysis (CKD stage 5D). CKD stage 3–4 was considered when an eGFR between 15 and 60 ml/min [25].

Patients were divided into 2 subgroups according to the time at the diagnosis of LN to compare the clinical, demographic and outcome variables by years. Group 1 consisted of paediatric-onset LN patients diagnosed and treated between the years 1993 and 2005, and group 2 between the years 2006 and 2023. The groups in the study were determined according to the time of access to biological treatments which became available in 2006 at our centres.

This study was approved by the Gazi University Institutional Review Board (IRB) (year 2023, approval no: 990).

### Statistical analysis

The statistical data were evaluated using the SPSS version 15.0 (SPSS Inc, Chicago, IL). Descriptive values were defined as number and percent. The Kolmogorov-Smirnov test was used to assess the normality of the distribution of continuous variables. According to the data distribution, the variables were specified as the mean ± s.d. or median [interquartile range (IQR)]. The differences between the two independent groups were evaluated by using the independent-sample *t* test for normally distributed variables, while the Mann–Whitney *U* test for non-normally distributed data. The *χ*^2^ test was used for the comparison of the categorical variables. The predictors for outcomes were analysed by the means of Cox proportional hazards ratio with 95% confidence intervals (CIs). Kaplan Meier analysis was used for the evaluation of survival rates. A *P*-value < 0.05 was accepted as statistically significant.

## Results

103 patients diagnosed with paediatric-onset SLE with kidney involvement were included in this study. Table 1 displays the main demographic characteristics and comparison of the patients who had been diagnosed and treated between the years 1993 and 2005 (group 1) and those between the years 2006 and 2023 (group 2). There were 28 (27%) patients in group 1, and 75 (73%) patients in group 2. In both groups, most patients were female (71% in group 1 vs. 79% in group 2, *P* = 0.439). The mean age at diagnosis of SLE was 14 (7) years in group 1, while 13 (5) years in group 2 (*P* < 0.001). The mean time to LN development and mean delay time to SLE diagnosis were significantly higher in group 1 (*P* = 0.049, and *P* = 0.004), whereas the mean age at onset of the first symptoms was significantly higher in group 2 (*P* < 0.001). Anaemia was significantly more common in group 1 (*P* = 0.020), while cardiovascular involvement in group 2 (*P* = 0.042). There were no significant differences in the other clinical signs related to SLE between the groups (*P* > 0.05). SLEDAI scores at first and last visits were found to be higher in patients with group 1 than in group 2 (*P* = 0.040 and *P* = 0.010, respectively). Furthermore, in the last visit, SLEDAI scores decreased in both groups.

**Table 1.** Demographics

	Group 1 (*n* = 28)		Group 2 (*n* = 75)		
Variable	*n* (%)	**Mean (** s.d.**) or Median (IQR)**	*n* (%)	**Mean (** s.d.**) or Median (IQR)**	*P*-value
Total	28 (100)		75 (100)		0.439
Male	8 (29)		16 (21)		
Female	20 (71)		59 (79)		
Mean age at onset of symptoms, years		12 (4.5)		12.5 (7.5)	**<0.001**
Mean age at diagnosis of SLE, years		14 (7)		13 (5)	**<0.001**
Mean age at diagnosis of LN, years		14 (7)		15.8 (13)	**<0.001**
Mean time to LN development, mo		1 (4)		0 (2)	**0.049**
Mean delay time to diagnosis, mo		5 (10)		2 (9.5)	**0.004**
Consanguineous marriage between parents	11 (39.2)		19 (25.3)		0.165
SLE signs					
Fever	9 (32.1)		29 (38.6)		0.541
Myositis	1 (3.5)		3 (4)		0.920
Arthritis	16 (57.1)		31 (41.3)		0.151
Alopecia	1 (3.5)		6 (8.8)		0.426
Mucosal ulcers	5 (17.8)		13 (17.3)		0.950
Cutaneous lupus	16 (55.1)		33 (44)		0.306
Neurologic involvement	5 (17.8)		5 (6.6)		0.087
Haematologic involvement	18 (64.2)		48 (64)		0.978
Anaemia	18 (64.2)		29 (38.6)		**0.020**
Leukopenia	5 (17.8)		18 (24)		0.505
Thrombocytopenia	7 (25)		30 (40)		0.158
Pulmonary involvement	6 (20.6)		12 (16)		0.570
Cardiovascular involvement	0 (0)		10 (13.3)		**0.042**
Disease activity					
SLEDAI (first visit)		27 (14–36)		16 (12–22)	**0.040**
SLEDAI (last visit)		9 (4–19)		4 (2–8)	**0.010**
Comorbid disease					
Polymyositis	1 (3.5)		0 (0)		0.100
ITP	0 (0)		2 (2.6)		0.382
Thyroiditis	0 (0)		1 (1.3)		0.539
Psoriasis	0 (0)		2 (2.6)		0.382
Type I DM	0 (0)		1 (1.3)		0.539
ARF	0 (0)		1 (1.3)		0.539
FMF	0 (0)		1 (1.3)		0.539

SLE: systemic lupus erythematosus; LN: lupus nephritis; mo: months; ITP: idiopathic thrombocytopenic purpura; DM: diabetes mellitus; ARF: acute rheumatic fever; FMF: familial Mediterranean fever; SLEDAI: systemic lupus erythematosus disease activity index.

Bold text highlights significant results.

The laboratory features and comparison of the patients are given in Table 2. CRP levels were higher and anti-phospholipid antibody positivity was more common in group 1 than in group 2 (*P* = 0.031 and *P* = 0.025, respectively). The remaining laboratory data did not show significant differences between the groups (*P* > 0.05).

**Table 2.** Laboratory features.

	Group 1 (*n* = 28)		Group 2 (*n* = 75)		
Variable	*n* (%)	Median (IQR)	*n* (%)	Median (IQR)	*P*-value
Laboratory findings (first visit)					
BUN (normal range: 5–18 mg/dl)		21.5 (13.5–34)		17.5 (11–24.7)	0.159
Creatinine (mg/dl)		0.8 (0.5–1.3)		0.6 (0.5–0.92)	0.238
Albumin (normal range: 3.5–5 g/dl)		3.6 (3–4.17)		3.1 (2.4–3.55)	0.155
C3 (normal range: 79–152 mg/dl)		69 (51–95)		53 (28–70)	0.220
C4 (normal range: 16–38 mg/dl)		10 (5–14)		6 (3–11)	0.096
CRP (normal range: 0–5 mg/l)		3.8 (2.4–15.3)		5 (1.5–15.7)	0.643
ESR (normal range: 0–20 mm/h)		78 (41–108)		49 (27–88)	0.114
Laboratory findings (last visit)					
BUN (normal range: 5–18 mg/dl)		19 (9–68)		13 (9–19)	0.360
Creatinine (mg/dl)		0.5 (0.45–1.42)		0.6 (0.5–0.7)	0.444
Albumin (normal range: 3.5–5 g/dl)		3.5 (2–4)		4.3 (3.95–4.6)	0.196
C3 (normal range: 79–152 mg/dl)		95 (69–111)		105 (76–128)	0.196
C4 (normal range: 16–38 mg/dl)		14 (11–18)		18 (11–26)	0.171
CRP (normal range: 0–5 mg/l)		3 (1.6–15.1)		1.1 (0.5–4.4)	**0.031**
ESR (normal range: 0–20 mm/h)		16 (9–38)		12 (5–29)	0.497
Autoimmune serology tests					
ANA positivity	23 (82.1)		69 (92)		0.149
Anti-dsDNA positivity	23 (82.1)		63 (84)		0.821
Anti-phospholipid antibody positivity	14 (50)		20 (26.6)		**0.025**
Direct coombs positivity	22 (78.5)		49 (65.3)		0.196
Urine tests					
Proteinuria^a^	21 (75)		60 (80)		0.581
Proteinuria (mg/day)		1225 (500–5700)		1785 (800–2985)	0.658
Haematuria	18 (64.2)		42 (56)		0.448

BUN: blood urea nitrogen; C3: complement 3; C4: complement 4; CRP: C-reactive protein; ESR: erythrocyte sedimentation rate; ANA: anti-nuclear antibody; anti-dsDNA: anti-double-stranded-deoxyribonucleic acid.

Bold text highlights significant results.

a Proteinuria was defined as >0.5 g/day.

Treatments of the patients with LN are given in Table 3. Corticosteroids were used in all patients, but pulse corticosteroid therapy at induction treatment was more common in group 2 than in group 1 (*P* = 0.009). While there was no difference between the groups for CYC in the induction phase (*P* = 0.558), AZA treatment was given more frequently in group 1 compared with group 2 as a maintenance treatment (*P* = 0.016). Since RTX treatment is a relatively new treatment modality that has been used in recent years, no patient in group 1 uses rituximab treatment. In contrast, it was used in 11 (14.6%) patients in group 2.

**Table 3.** Treatments.

	Group 1 (*n* = 28)	Group 2 (*n* = 75)	
Variable	*n* (%)	*n* (%)	*P*-value
Treatment strategies			
Induction treatment			
Corticosteroid	28 (100)	75 (100)	0.463
Pulse corticosteroid	21 (75)	70 (93.3)	**0.009**
CYC	20 (71.4)	49 (65.3)	0.558
MMF	0 (0)	26 (34.6)	**0.000**
Anti-CD20			
RTX	0 (0)	11 (14.6)	**0.032**
Plasmapheresis	0 (0)	10 (13.3)	**0.042**
Maintainence			
Corticosteroid	25 (89.2)	73 (97.3)	0.090
Pulse corticosteroid	7 (25)	17 (22.6)	0.803
MMF	10 (35.7)	30 (40)	0.350
AZA	14 (50)	19 (25.3)	**0.016**
Anti-CD20			
RTX	0 (0)	10 (13.3)	**0.042**
Anti-hypertensive treatment			
ACE inhibitor	14 (50)	28 (37.3)	0.244
Others	7 (25)	9 (12)	0.105

CYC: cyclophosphamide; MMF: mycophenolate mofetil; ACE: Angiotensin-converting enzyme.

Bold text highlits significant results.

The features of kidney involvement are shown in Table 4. In both groups, the majority of the patients had proliferative nephritis (class III and/or class IV) (53.5% in group 1 vs. 68% in group 2). Nephrotic syndrome was significantly more common in group 1 than in group 2 (*P* = 0.044). When the outcome of LN was evaluated, complete remission was significantly more common in group 2 (*P* = 0.005), while ESKD and death in group 1 (*P* = 0.005 and *P* = 0.001, respectively). Among overall patients with paediatric-onset LN, 9 (8.7%) patients had ESKD, and 6 (5.8%) patients died during follow-up. Serious infections were the reason for the death of patients.

**Table 4.** Kidney involvement.

	Group 1 (*n* = 28)	Group 2 (*n* = 75)	
Variable	*n* (%)	*n* (%)	*P*-value
Kidney involvement			
Nephritic features	6 (21.4)	22 (29.3)	0.422
Nephrotic syndrome	15 (53.5)	24 (32)	**0.044**
Nephritic+nephrotic features	5 (17.2)	10 (13.3)	0.610
Hypertension	9 (32.1)	25 (33.3)	0.909
Kidney bx classification			
Class I	0 (0)	2 (2.6)	0.382
Class II	8 (28.5)	10 (13.3)	0.070
Class III	3 (10.7)	9 (12)	0.856
Class IV	12 (42.8)	42 (56)	0.234
Class V	5 (17.8)	12 (16)	0.821
Outcome			
Complete remission	4 (14.2)	33 (44)	**0.005**
Partial remission	8 (28.5)	32 (42.6)	0.191
CKD stage 3–5D	5 (17.8)	6 (10.6)	0.149
ESKD	6 (21.4)	3 (4)	**0.005**
Death	5 (17.8)	1 (1.3)	**0.001**

CKD: chronic kidney disease; ESKD: end-stage kidney disease; bx: biopsy.

Bold text highlights significant results.

**Figure 1.**
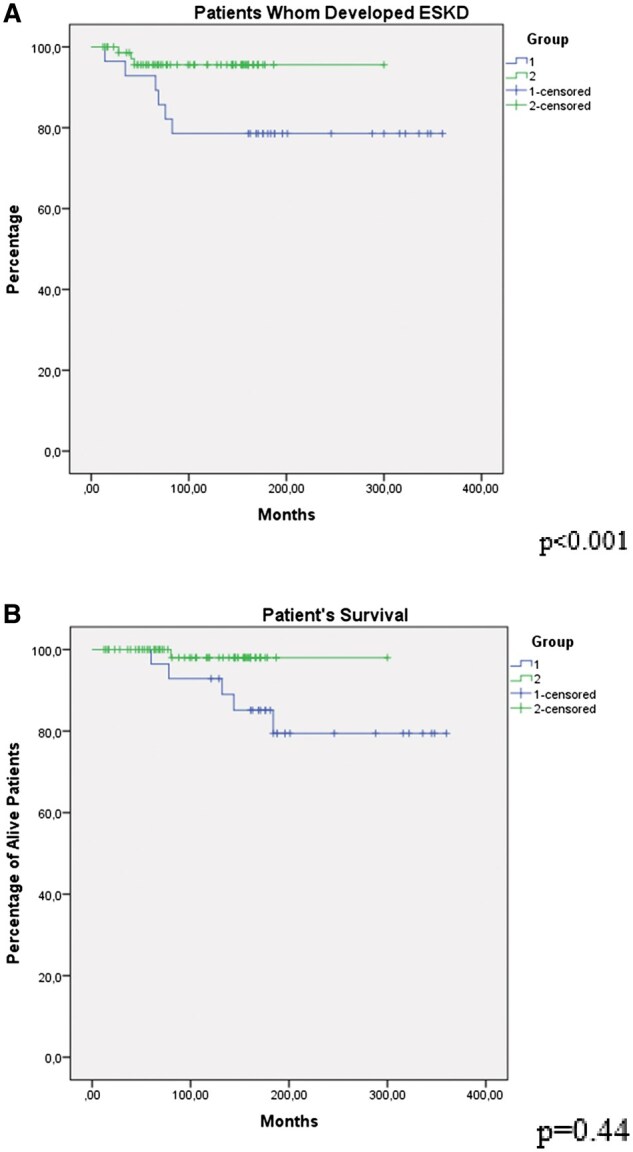
Kaplan Meier survival analysis regarding ESKD and death. There was a significantly better outcome for the development of ESKD in Group 2 compared to Group 1 (*P* < 0.001) (A), and patient survival was not different between the two groups (*P* = 0.44) (B)

Primary outcome measures were ESKD and death. Kidney survival analysis of the groups revealed a significantly better outcome for the development of ESKD in group 2 compared with group 1 (*P* < 0.001) (Fig. 1A), while patient survival was not different between the two groups (*P* = 0.44) (Fig. 1B). 10-year ESKD-free survival was 79% in group 1 and 96% in group 2 (Time to ESKD per patient was 14, 35, 66, 69, 76, 83 months in group 1, and 28, 41, 44 months in group 2, respectively).

There were no associations between ESKD and gender, age at diagnosis of SLE and LN, delay time to diagnosis, ANA positivity, anti-dsDNA positivity, anti-phospholipid positivity or direct coombs positivity (*P* > 0.05, for each). Proteinuria and SLEDAI scores at the first visit were independent risk factors for ESKD (*P* = 0.037 and *P* = 0.024) (Table 5).

**Table 5.** The cox regression analysis to evaluate the predictors of ESKD

Variable	HR (95% CI)	*P*-value
Gender	0.383 (0.07–2.093)	0.268
Age at diagnosis of SLE	0.961 (0.885–1.043)	0.342
Age at diagnosis of LN	1.02 (0.944–1.102)	0.612
Delay time to diagnosis	1.045 (0.988–1.104)	0.122
BUN	0.139 (0.003–0.187)	0.550
Creatinine	0.142 (0.110–0.219)	0.510
C3	0.104 (0.002–2.208)	0.536
C4	0.096 (0.011–3.259)	0.567
CRP	0.040 (0.020–0.055)	0.777
ESR	0.177 (0.001–0.314)	0.304
Proteinuria	3.330 (0.084–5.436)	**0.037**
ANA positivity	1.108 (0.263–4.671)	0.889
Anti-dsDNA positivity	0.941 (0.292–3.029)	0.918
Anti-phospholipid antibody positivity	0.801 (0.736–2.407)	0.197
Direct coombs positivity	0.571 (0.46–2.363)	0.471
Hypertension	0.558 (0.117–1.207)	0.580
Class III and/or class IV nephritis	0.165 (0.128–1.198)	0.667
SLEDAI at first visit	4.651 (1.351–9.541)	**0.024**
Pulse corticosteroid treatment	1.115 (0.66–1.246)	0.250
Plasmapheresis	0.937 (0.749–1.143)	0.590
RTX treatment	0.925 (0.75–1.124)	0.524

SLE: systemic lupus erythematosus; LN: lupus nephritis; BUN: blood urea nitrogen; C3: complement; C4: complement 4; CRP: C-reactive protein; ESR: erythrocyte sedimentation rate; SLEDAI: Systemic Lupus Erythematosus Disease Activity Index.

Bold text highlights significant results.

## Discussion

In this study, we evaluated demographic, laboratory, and treatment data of paediatric-onset LN patients who had been diagnosed during the last three decades and compared all data belonging to patients diagnosed before and after 2006. We showed that patients with paediatric-onset LN had improved kidney outcomes and SLEDAI scores due to better management with new therapeutic agents over the recent years.

Female predominance did not change. Moroni *et al.* described 499 adult patients with LN who were followed up between 1970 and 2016 years, divided into three groups according to the years of follow-up; group 1 1970–1985, group 2 1986–2001 and group 3 2002–2016, and they reported a higher age at diagnosis of SLE and LN in patients who followed between 1970 and 1985 [16]. In our study, age at diagnosis of SLE and LN, and delay time to diagnosis of SLE were significantly higher in patients who followed up at an earlier time. We suggest that earlier diagnosis of paediatric-onset SLE resulted from increased awareness by the increased number of rheumatology and nephrology centres in our country and easy access to these centres might have decreased lag time in the diagnosis of SLE in recent years.

SLE is an autoimmune multi-systemic disease, so we evaluated other system involvements in addition to LN. Anaemia was more common in patients in group 1, those diagnosed before 2006, while cardiovascular involvement was more common in patients who were followed up in recent years. SLEDAI scores at the first visit and the last visit were higher in group 1 than those followed up in recent years. Although group 1 had higher disease activity based on SLEDAI score (21 vs. 19) at diagnosis, the decrease in SLEDAI score at the last visit was more striking in group 2, which may be attributable to the close follow-up and the success of newer treatment options.

Moroni *et al.* reported lower levels of creatinine and albumin, whereas a higher level of C4 in patients with LN who were followed up in 1970–1985 than those in 2002–2016 [16]. In our study, higher CRP and more common antiphospholipid antibody positivity were observed in group 1, and no significant differences were found between the groups in terms of creatinine and serum albumin levels.

Proliferative LN, including class III and class IV, has the most aggressive course and the poorest kidney outcome compared with non-proliferative LN [7]. In children with LN, the rates of proliferative LN are as high as 70–90% [6, 7, 18, 26–28]. In our cohort, 64% of the paediatric-onset SLE patients had proliferative LN, and there was no difference between the two time periods. Risk factors for the severity and outcome of LN were described as ethnicity, histopathological classification, male gender, hypertension, high disease activity, decreased kidney function at baseline, treatment response and disease relapse [6, 7, 29, 30]. Recently, Park *et al.* described male gender and failure to disease remission achievement in the first year of the treatment as the predictors of CKD [31], while Qiu *et al.* reported hypertension, nervous system involvement, non-compliance to the treatments, and lower GFR level at disease-onset were independent risk factors for poor disease outcome of paediatric-onset LN [32]. Improvements in the prognosis of paediatric LN patients with effective suppression of active disease and good medication adherence during long follow-up periods were reported from large paediatric cohorts [27, 28]. In our study, higher disease activity and proteinuria levels at baseline were predictors of ESKD. Qiu *et al.* reported a 5-year ESKD-free rate of 97% in 220 paediatric LN patients followed up between 2012 and 2018 [32]. Singh *et al.* described that renal survival was 96%, 89% and 78% at 1, 5 and 10 years, respectively, on paediatric LN patients followed up between 1991 and 2013 [33]. In our study, 10-year ESKD-free survival was 79% in group 1 and 96% in group 2. As expected, we also showed that ESKD and death were lower, and complete kidney remission was higher in group 2 compared with group 1, probably due to better management and newly developed treatments. Disease activity scores are important to decide the intensity of immunosuppressive treatment in paediatric rheumatic diseases [22, 34], and we think that paediatric-onset LN who have higher disease activity at the baseline should be treated intensively to suppress disease activity earlier and prevent damage to vital organs. Treatment plans in paediatric-onset SLE are mainly based on adult trials due to the lack of randomized controlled trials in children [34]. Treatment of class III-IV LN consists of induction and maintenance periods. In induction therapy, corticosteroids are the main treatment option, and mostly pulse corticosteroid treatment is prescribed at a dose of 15–30 mg/kg/day (max 1 g/day) for 3–5 days, followed by 1–2 mg/kg/day prednisolone (max 60 mg/day) with a gradual tapering. In the treatment of proliferative LN corticosteroids and cDMARDs are commonly used, despite their significant side effects [12, 35]. Many studies demonstrated no differences between CYC and MMF treatment in induction therapy in the childhood proliferative LN [6, 36, 37]. All patients with paediatric-onset SLE should be prescribed HCQ [1]. RTX is a chimeric monoclonal antibody that inhibits B cell development due to binding CD20 antigen and has recently been used as an effective treatment option during induction and/or maintenance period in patients with organ-threatening manifestations or in treatment-resistant cases [38–40]. Recently, Chan *et al.* described the utility of RTX treatment on paediatric-onset LN patients who had life-threatening and/or did not respond to standard immunosuppressive treatments [38]. Plasmapheresis is an invasive treatment procedure through removing immune complexes from circulation in the presence of life-threatening organ involvement in paediatric-onset SLE, antiphospholipid syndrome or vasculitis [41]. We performed plasmapheresis in the second period in patients with LN because these patients also had severe life-threatening SLE-related organ involvement, such as concomitant neuropsychiatric involvement, alveolar haemorrhage, or thrombotic microangiopathy. Belimumab is a monoclonal antibody against soluble B lymphocyte stimulator and was successfully used in paediatric-onset SLE case series to decrease disease activity and also help tapering corticosteroids [12, 42]. However, we did not use belimumab treatment in our patients because it is not yet approved in paediatric-onset SLE in our country. Both RTX and belimumab succeeded in reducing daily corticosteroid dosage and provided better disease control with an acceptable safety profile in paediatric-onset SLE [39]. Enalapril, an angiotensin-converting enzyme inhibitor, should be given to patients who have proteinuria and/or hypertension [1].

There are limitations to our study: (i) Retrospective study design. (ii) Anti-extractable nuclear antigen (ENA) test was not available in some patients. (iii) Detailed kidney biopsy results were not available, therefore, we could not calculate activity and chronicity indices. On the other hand, the assessment of long-term outcomes in a large cohort of paediatric-onset LN patients and compared treatment regimens are the main strengths of this study.

In conclusion, earlier diagnosis through an increased number of paediatric rheumatology and paediatric nephrology centres, and better management of disease due to close follow-up and new treatment options such as biologic DMARDs provided a better kidney outcome in patients with paediatric-onset SLE during the last 15 years compared with the earlier era. Additionally, high levels of proteinuria with high disease activity scores should alert clinicians about a potential kidney disease progression.

## Supplementary Material

keaf151_Supplementary_Data

## Data Availability

The data underlying this article will be shared on reasonable request to the corresponding author.
