# Supplementary material for: How have the clinical, laboratory, treatment features and outcomes in children with lupus nephritis progressed over the last 30 years?
Source: Rheumatology (Oxford). 2025 Mar 19;64(7):4275–82. doi: 10.1093/rheumatology/keaf151 (PMC12212907; doi:10.1093/rheumatology/keaf151)
Supplement: keaf151_Supplementary_Data [file keaf151_supplementary_data.docx]

**Supplementary figure S1. The inclusions and exclusions of the study with a workflow chart.**

2 patients were excluded due to follow up time less than 6 months.

Finally, 103 pediatric patients with lupus nephritis were included to the study.

Medical records of 111 pediatric patients with lupus nephritis were evaluated.

6 patients were excluded from the study due to the lack of medical data fully.
